# Influence of Chloride Concentration on Stress Corrosion Cracking and Crevice Corrosion of Austenitic Stainless Steel in Saline Environments

**DOI:** 10.3390/ma13245640

**Published:** 2020-12-10

**Authors:** Chun-Ping Yeh, Kun-Chao Tsai, Jiunn-Yuan Huang

**Affiliations:** Institute of Nuclear Energy Research (INER), 1000 Wenhua Rd., Longtan District, Taoyuan City 32546, Taiwan; tsaijohn@iner.gov.tw (K.-C.T.); jyhuang@iner.gov.tw (J.-Y.H.)

**Keywords:** chloride concentration, crevice corrosion, relative humidity, stainless steel, stress corrosion cracking

## Abstract

Stainless steels are used as canister materials for interim storage of spent fuel. Crevice corrosion has proved to be a safety concern of 304L stainless steel spent fuel canisters, when exposed to the saline environments of coastal sites. To study the effects of chloride concentration and test duration on the crevice corrosion behavior, and the effect of relative humidity on the initiation of discrete SCC cracks, a test program was conducted on the 304L steel specimens sprayed with synthetic sea water of 3.5 wt.%. The salt-deposited specimens, wrapped up with a crevice former to form a crevice configuration, were then exposed to an environment at 45 °C with a pre-set 45%, 55%, and 70% relative humidity (RH), for 400 h and 10,000 h, respectively. The surface features and crack morphology of the tested 304L stainless-steel specimens were examined by energy-dispersive spectrometry (EDS) and electron back scatter diffraction (EBSD). For the specimens deposited with a chloride concentration of 1 g/m^2^, no cracks were found in the corroded regions after 400-h exposure, whereas SCC cracks were observed with the specimens tested for 10,000 h at all three pre-set relative humidity. The specimens tested at the pre-set relative humidity 45% are characterized with discrete SCC cracks, but, on the other hand, those exposed to the environments of 55% and 70% relative humidity show SCC cracks of distinct features. From the results of 10,000-h tests, it is inferred that the chloride concentration threshold for SCC initiation of 304L stainless steel at 45 °C is between 0.1 g/m^2^ and 1 g/m^2^.

## 1. Introduction

Spent nuclear fuel, when discharged from reactors, is first stored in the spent fuel pool to cool down fuel temperature and radioactivity. Prior to final disposal, spent nuclear fuels are loaded and stored in the austenitic stainless-steel canister of the dry cask storage system. The stainless-steel canister is filled with helium gas to facilitate spent fuel decay heat removal by natural convection. Therefore, the surface of canister is exposed to ambient air. In Taiwan, the dry cask storage facilities for spent fuel are located in coastal regions. Sea salt could deposit on the canister surface by an airborne process or seawater spray. Stainless steels canisters are well-known to be susceptible to atmospheric localized corrosion in the presence of chloride ions [1,2,3,4,5]. The dry cask storage systems, as planned, are to operate for about 40–60 years [6]. Therefore, the integrity of the canisters must be maintained to ensure the spent fuel interim storage safety.

Stainless steels which with passive film on its surface have excellent corrosion resistance, and are often used in some aggressive environments. Nevertheless, austenitic stainless steels are inclined to suffer from localized corrosion, especially in the presence of crevice sites on the surfaces [7]. Crevice corrosion is a corrosion on metal surfaces at the gap between two surfaces, and the mass transfer process was restricted due to the narrow crevice configuration. It is inferred that crevice corrosion behaviors of stainless steel are associated with the evolution process of the passive film which inside crevice [8]. With increasing Cl^−^ ions concentration, the passive film is more unstable, and it leads to more severe crevice corrosion [9].

For crevice corrosion, by means of trapping chloride deposits on the surface of canister, it can be an essential factor of atmospherically-induced stress corrosion cracking (AISCC). The initiation and propagation of crevice corrosion are highly dependent on the temperature, aggressive ions (e.g., Cl^−^) and crevice geometry. In the horizontal and vertical canister systems, the configuration of crevice is formed where the canister contacts with the support structure of storage module [10].

As for stainless steel, stress corrosion cracking (SCC) is susceptible in aggressive environments, for instance, the SCC caused by chlorides and particles of sea salt. At crevice sites, the increase of corrosion products volume could result in local stresses to prompt the happening of stress corrosion cracking. Moreover, Tani et al. reported that when crevice corrosion occurs, it will contribute to initiation of stress corrosion cracking on the surfaces beneath particles of sea salt [11].

Crevice corrosion behavior can be explained by two common mechanisms: Critical crevice solution (CCS) and IR drop mechanism [8]. As far as the critical crevice solution mechanism is concerned, it indicates that the depletion of oxygen within the crevice could cause the acidification of solution at crevice sites, which result in the breakdown of passive film and initiation of crevice corrosion. This theory accentuates the accumulation of aggressive ions (e.g., Cl^−^) at crevice sites and then the depassivation followed by base metal dissolution [12,13]. As far as the IR drop mechanism is concerned, crevice corrosion takes place by potential transition from passive state to active state, when the value of IR exceeds a critical threshold. The IR drop involves the value of potential drop which is obtained by multiplication of the resistance of the electrolyte and the current at crevice sites [14,15]. Aggressive ions (e.g., Cl^−^) encourage the crevice corrosion by raising the corrosion current, resulting in an increase of the IR value [16,17].

Scatigno and coworkers reported the influence of deposited salt on chloride-induced stress corrosion cracking in 304L steel at 90 °C with 70% RH. They suggested that atmospherically-induced stress corrosion cracking was occurred in the 1.7 g/m^2^ MgCl_2_ chloride concentration [18]. Shoji and coworkers presented a research for the atmospherically-induced stress corrosion cracking phenomenon with regard to relative humidity, temperature, and chloride ions, and demonstrated it happening in type 316L and 304L steels under ambient situations. It is inferred that MgCl_2_ is the main sea-salt constituent for the promotion of low temperature atmospherically-induced stress corrosion cracking in type 316L and 304L steels [19]. For the corrosivity of chloride deposition on the canister surface under the given exposure ambient conditions, it was reported in the following descending order: CaCl_2_ > MgCl_2_ > NaCl. Apparently, it was controlled by the concentration of chloride on surfaces, due to the interaction of a given amount of salt with water vapor in the air [20]. In a summary of investigations on AISCC phenomena of stainless steels, Engelberg et al. summarized a susceptibility order for occurrence of AISCC of 304L steels. It was provided that the relative humidity ranges which atmospherically-induced stress corrosion cracking occurred in the 25 g/m^2^ MgCl_2_ chloride concentration, based on the experimental results of Shoji et al. [21]. Padovani et al. suggested that 316L stainless steel is susceptible to AISCC at 30 °C under MgCl_2_ deposition [22]. Moreover, the critical RH for type 304L steel SCC initiation after 5000 h experiments with chloride concentration 1 g/m^2^ is between 45% and 55% [23].

Masuda and coworkers illustrated the SCC behavior of the 304 stainless steel in terms of the pit growth, slip deformation and surface potential distribution. SCC tests were done at 28% RH and 343 K with MgCl_2_ droplets on the specimen. Discontinuous cracks were often observed near the crack tip [24]. Based on the observations on the SCC phenomenon of 321 stainless steel in an MgCl_2_ solution, Qiao and coworkers suggested that the interaction between discontinuous microcracks and main crack could enhance the effective stress intensity factor and promote crack coalescence, leading to mechanical fracture of the ligaments between the cracks in stainless steel [25].

The goal of the study is to evaluate the behavior of crevice corrosion for type 304L steel under different conditions of chloride concentrations, and test duration. Moreover, the effect of relative humidity on the initiation of discrete SCC cracks was also examined in this research to have a better understanding of the initiation of SCC at the sites of crevice corrosion under different relative humidity.

## 2. Experimental Procedure

**Figure 1:**
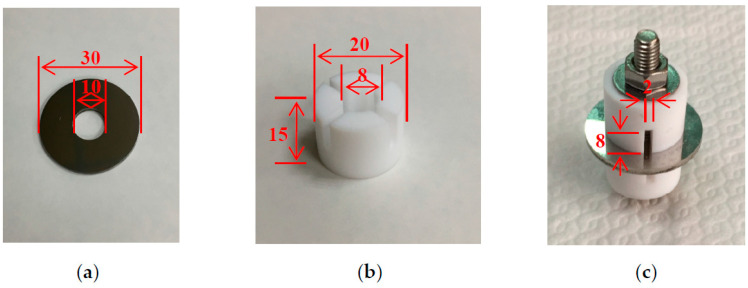
Image of the specimen and the crevice former used for crevice corrosion experiment. (**a**) Specimen, (**b**) crevice former, and (**c**) assembled image. (Unit: mm).

Figure 1a,b present the dimensions of the crevice former and the test specimen and, respectively, and an assembled image of a test specimen and a crevice former is shown in Figure 1c. The thickness of the test specimen is 3 mm. The chemical compositions of 304L stainless steel used in this research are listed in Table 1. Specimens were manufactured from type 304L steel, and the material of crevice former was polytetrafluoroethylene (PTFE). Duplicate tests were conducted for each experimental condition. Before crevice corrosion test, the specimen surfaces were ground with 2000-grit abrasive paper.

The chemical compositions of sea salt used in this study, based on ASTM D 1141-98 (13) Formula a, Table X1.1, Sec.6, are listed in Table 2. The specimens were firstly sprayed with 3.5 wt.% synthetic sea water and then dried on the hot plate at 60 °C for 15 min. After the specimens were dried, the mass change of the specimens was evaluated for the calculation of chloride concentration in g/m^2^. The specimen chloride concentration was at one of the pre-determined levels of 0.1 and 1 g/m^2^. The specimen and the crevice former were fixed with a M6 screw by applying a torque of 1.13 N m to form a crevice configuration. The constant-temperature/humidity chambers were kept at a combination of the ambient temperature of 45 °C with a RH = 45%, 55%, and 70%, for 400 h and 10,000 h, respectively. Samples for microstructural inspection were obtained through a metallographic process of sample preparation. The specimens were washed ultrasonically in deionized (DI) water and then dried cautiously and mounted in the resin. The mounted samples were polished by using Aluminum oxide powder. Surface features and microstructures of the tested specimens were examined with an optical microscope and a scanning electron microscope (SEM). The technique of energy-dispersive spectrometer (EDS) is widely used for providing semi-quantitative analysis of materials. In this study, the crack regions were selected for the EDS mapping analysis. The elemental distributions at crack regions were fully mapped by collecting a full EDS spectrum at each pixel for the area of the crack region. The technique of EDS was also used to determine the compositions of the corrosion bands. Furthermore, a SEM equipped with an electron backscatter diffraction (EBSD) detector was employed to examine the SCC features of the specimens. In addition, the kernel average misorientation (KAM) map for the tested specimens can be obtained by inputting the EBSD map into the software for further data processing. The crack morphology of the specimens was characterized with an SEM.

## 3. Results and Discussion

### 3.1. Analysis of Surface Morphology

**Figure 2:**
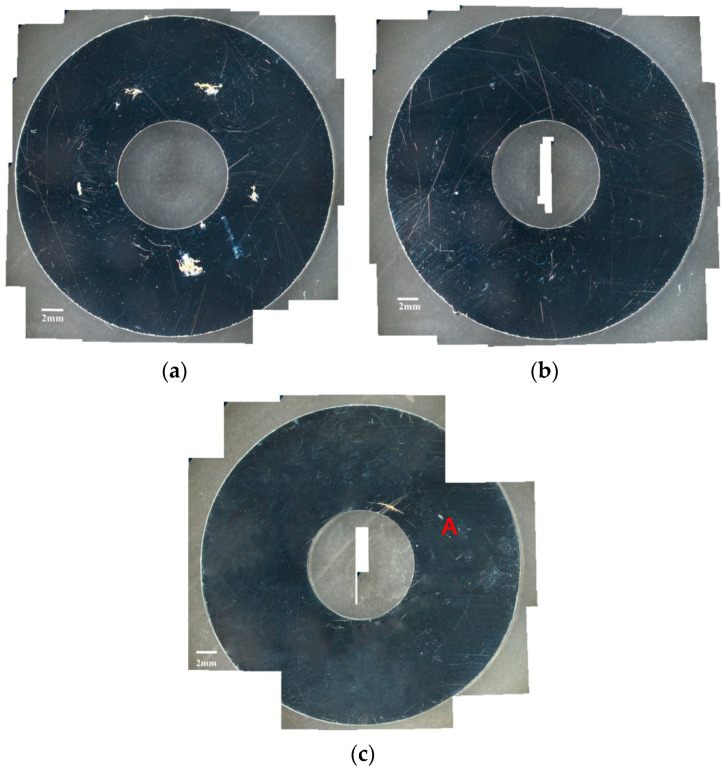
Macrographs of the corroded regions of the specimens with chloride concentration of 0.1 g/m^2^ after 400-h testing at: (**a**) Relative humidity (RH) = 45%, (**b**) RH = 55%, and (**c**) RH = 70%.

**Figure 3:**
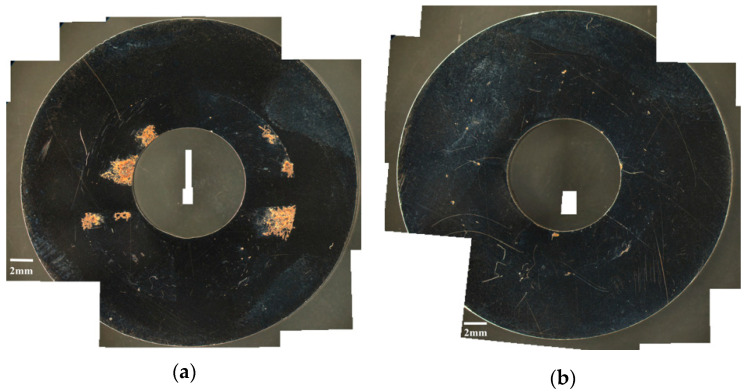

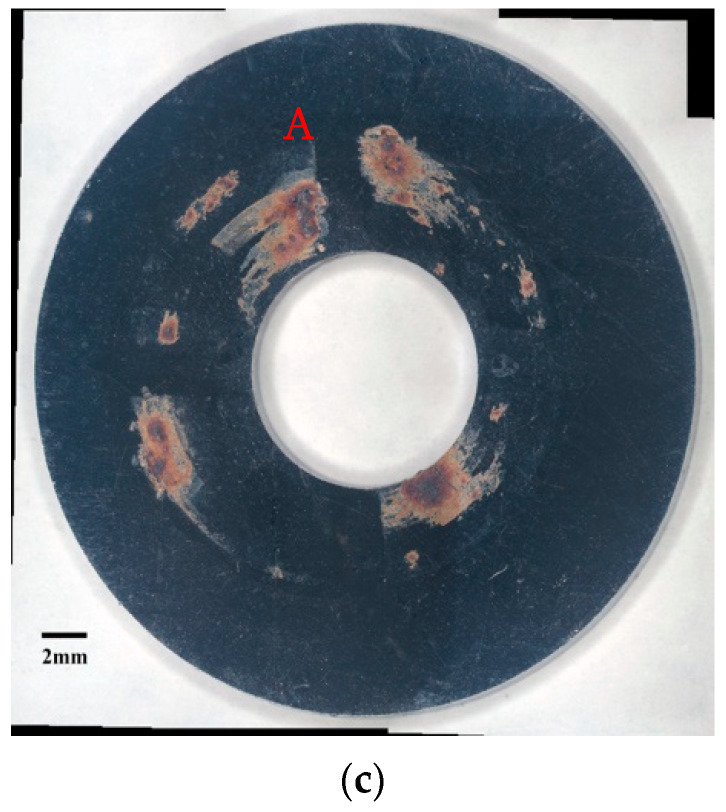
Macrographs of the corroded regions of the specimens with chloride concentration of 0.1 g/m^2^ after 10,000-h testing at: (**a**) RH = 45%, (**b**) RH = 55%, and (**c**) RH = 70%.

**Figure 4:**
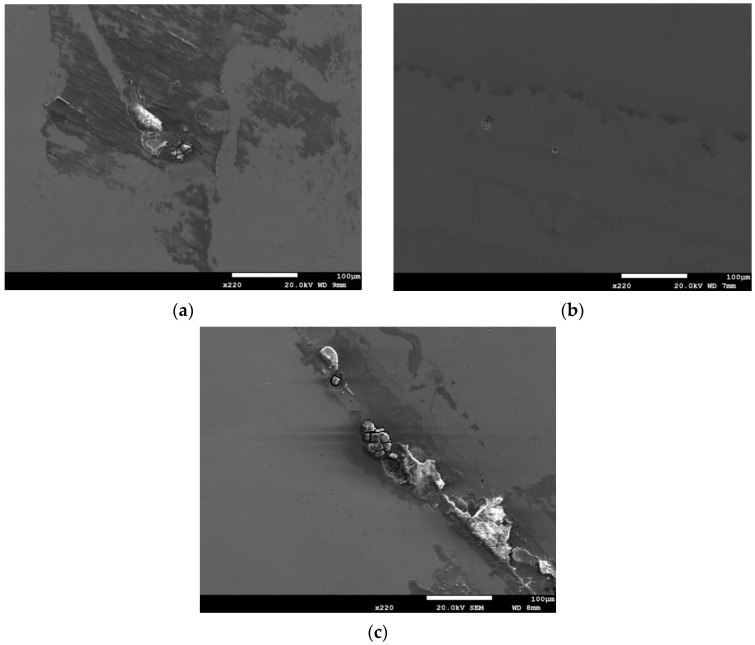
SEM micrographs of the specimens with chloride concentration of 0.1 g/m^2^ after 400-h testing at: (**a**) RH = 45%, (**b**) RH = 55%, and (**c**) RH = 70%.

**Figure 5:**
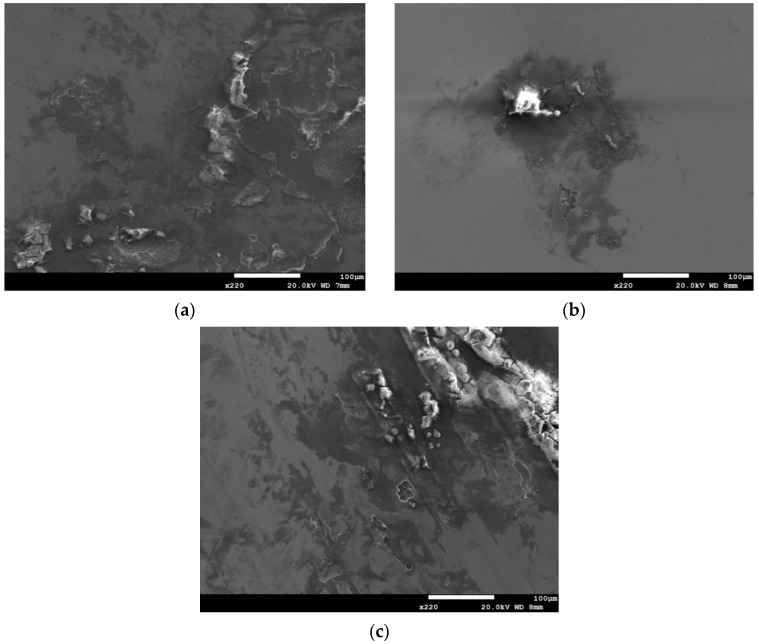
SEM micrographs of the specimens with chloride concentration of 0.1 g/m^2^ after 10,000-h testing at: (**a**) RH = 45%, (**b**) RH = 55%, and (**c**) RH = 70%.

Figure 2 and Figure 3 are the macrographs of the 304L steel specimens with chloride concentration of 0.1 g/m^2^ after testing for 400 and 10,000 h at three different relative humidity levels, respectively. A trace of crevice corrosion was discerned with the specimens after 400-h testing. Significant crevice corrosion was observed with the specimens tested for 10,000 h. As illustrated in Figure 2a–c and Figure 3a–c, the rusted areas of both 400-h and 10,000-h tested specimens decrease with increasing the relative humidity from 45% RH to the least at 55% RH, but then increase at 70% RH; the rusted areas of the specimens tested for 10,000 h at 70% RH are clearly larger than those tested at 45% and 55% RH. Since the chloride concentration of 0.1 g/m^2^ is much lower than those reported in the literature [19,20,21], the local chloride concentration may be reduced due to the higher relative humidity of 55% compared to the relative humidity of 45%, leading a limited concentration of chloride transported to the crevice sites of the specimens. As a result, it shows a tendency for a decrease in the corrosion effect when the humidity goes from 45% to 55%. However, due to a low chloride concentration of the specimens tested, it is necessary to have a higher relative humidity environment to facilitate the transportation of a sufficient amount of chlorine (Cl) to the crevice sites for the initiation of corrosion. When the humidity goes from 55% to 70%, the effect of higher relative humidity has an advantage over the effect of chlorine dilution, resulting in an increase in the rusted areas. It is hypothesized that the rusted areas are influenced by a synergy effect of relative humidity and chlorine dilution. Moreover,** the deliquescence relative humidity (DRH) of NaCl at 45 °C is about 73% [26], which is close to the relative humidity of 70% used in this work. With the possible additional NaCl deliquescence, it is inferred that the corrosion effect may be enhanced, leading to the reduction of chlorine dilution effect at RH = 70%. Figure 4 and Figure 5 respectively present the SEM micrographs of the specimens with chloride concentration of 0.1 g/m^2^ after 400-h and 10,000-h testing at three different relative humidity levels. Rust on the specimens shows evidence of crevice corrosion induced by chloride. The morphologic features of crevice corrosion vary with relative humidity. Figure 4a,c reveal that there are some rust spots on the specimens tested for 400 h, and that shallow corrosion exists beneath the rust spots. Compared to Figure 4a,c for those exposed to 45% and 70% RH, the corroded areas of the specimens tested at 55% RH are smaller, Figure 4b. As with the 400-h tested specimens, Figure 4a,c, the specimens tested for 10,000 h show some rusted spots, beneath which there exists shallow corrosion, as exemplified in Figure 5a,c. And the corroded areas on the specimens exposed to 55% RH, Figure 5b, are smaller, relative to those shown in Figure 5a,c for the specimens tested at 45% and 70% RH. Moreover, no cracks were found with all the specimens tested for 400 and 10,000 h at three different relative humidity levels, Figure 4 and Figure 5. In relative terms, the corroded areas on the 10,000-h tested specimens are larger than those on the corresponding specimens tested for 400 h.

**Figure 6:**
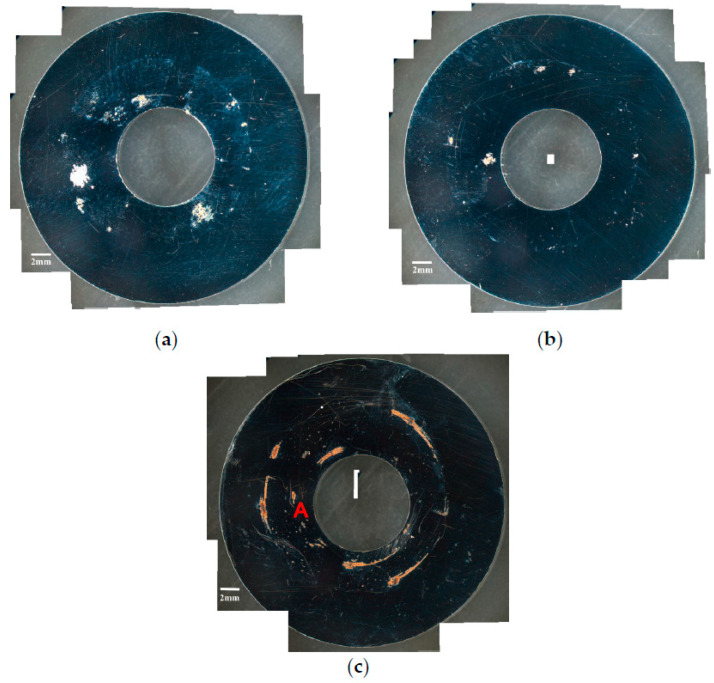
Macrographs of the specimens with 1 g/m^2^ chloride concentration after 400-h testing at: (**a**) RH = 45%, (**b**) RH = 55%, and (**c**) RH = 70%.

**Figure 7:**
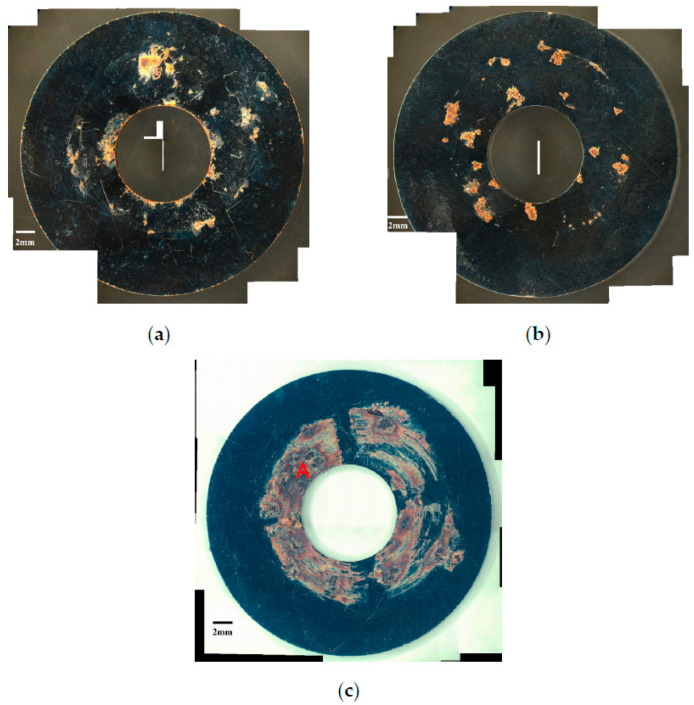
Macrographs of the specimens with 1 g/m^2^ chloride concentration after 10,000-h testing at: (**a**) RH = 45%, (**b**) RH = 55%, and (**c**) RH = 70%.

**Figure 8:**
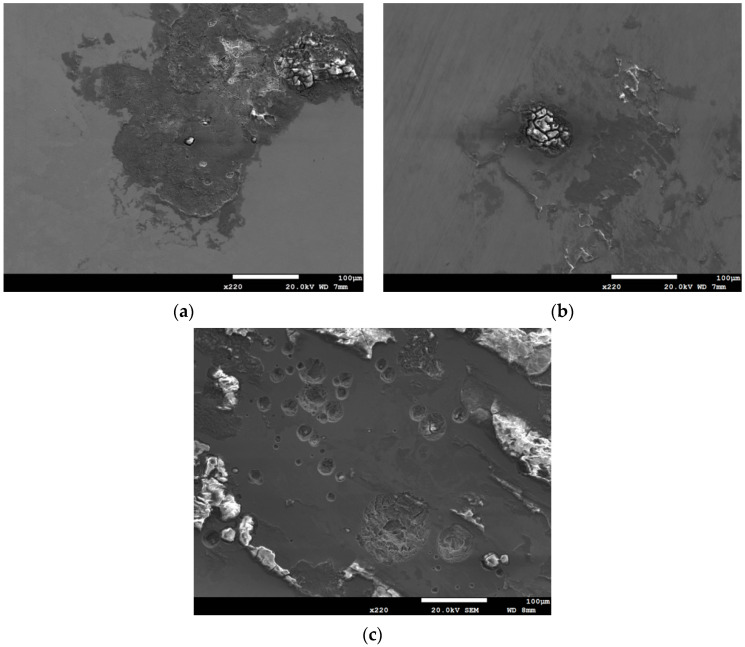
SEM micrographs of the specimens with 1 g/m^2^ chloride concentration after 400-h testing at: (**a**) RH = 45%, (**b**) RH = 55%, and (**c**) RH = 70%.

**Figure 9:**
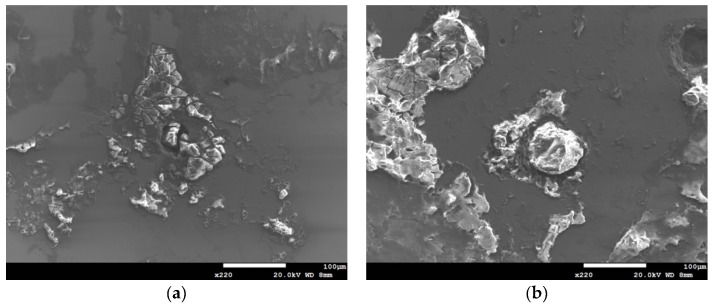

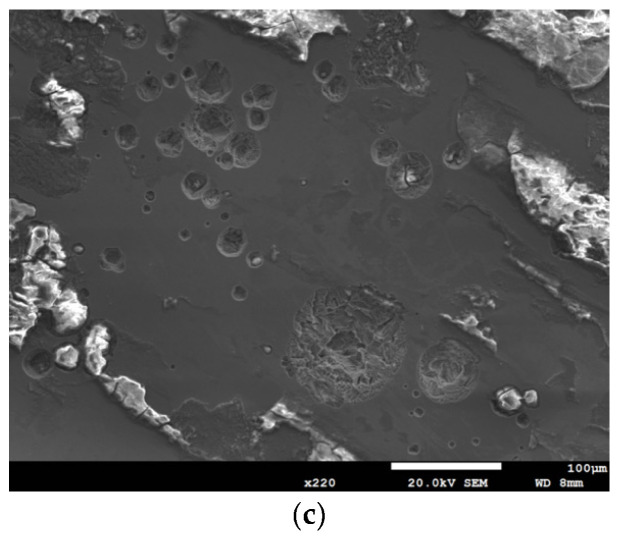
SEM micrographs of the specimens with 1 g/m^2^ chloride concentration after 10,000-h testing at: (**a**) RH = 45%, (**b**) RH = 55%, and (**c**) RH = 70% with chloride concentration of 1 g/m^2^.

Figure 6 and Figure 7 are the macrographs of the 304L austenitic stainless steel specimens deposited with 1 g/m^2^ chloride concentration after 400-h and 10,000-h testing at three different relative humidity levels, respectively. Crevice corrosion was observed with the specimens after 400-h testing. The rusted areas on the specimens tested for 400 h decrease with increasing the relative humidity from 45% RH to a minimum at 55% RH, but then increase at RH = 70%, as shown in Figure 6. A comparison of Figure 7a–c illustrates that there is little difference in the rusted areas on the specimens after testing for 10,000 h under the RH = 45% and 55%, but that the areas of rusted increase significantly at 70% RH. Furthermore, for the 10,000-h tests, the specimens with 1 g/m^2^ chloride deposit, Figure 7, show more serious corrosion at all three relative humidity levels than those with 0.1 g/m^2^ chloride deposit, Figure 3. Figure 8 and Figure 9 show the SEM micrographs of the specimens deposited with 1 g/m^2^ chloride tested at different relative humidity levels for 400 and 10,000 h, respectively. As with the specimens with 0.1 g/m^2^, rust on the specimens with 1 g/m^2^ is evidence of crevice corrosion induced by chloride. Figure 8a,b reveal some rust spots present on specimens. It also illustrates shallow corrosion beneath rust spots. In addition to rust spots, Figure 8c illustrates larger corrosion pits on the specimens exposed to 70% RH, compared to the corrosion pits shown in Figure 8a,b for the specimens tested at 45% and 55% RH. No cracks were found with the specimens deposited with 1 g/m^2^ chloride after testing for 400 h at three different relative humidity levels, as exemplified in Figure 8. The specimens after 10,000-h testing were severely corroded. The specimens exposed to 70% RH, Figure 9c, appear more severely corroded than those subjected to 45% and 55% RH of Figure 9a,b. Figure 9 shows more rust spots, shallow corrosion beneath the rust spots, and corrosion pits.

**Figure 10:**
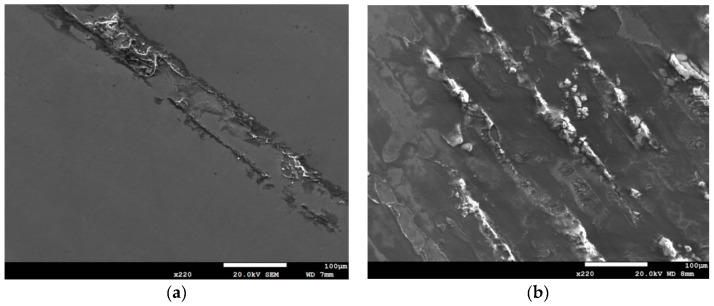

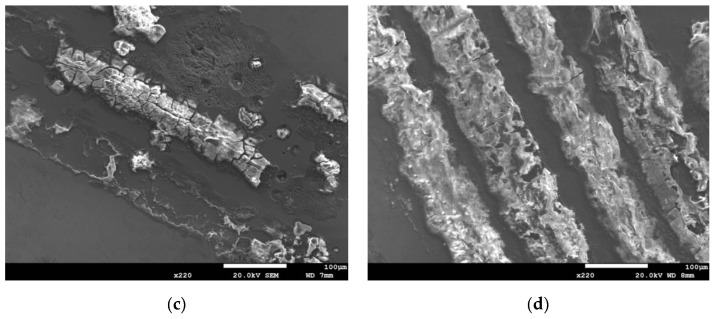
SEM micrographs of corrosion bands of specimens deposited with: 0.1 g/m^2^ chloride (**a**) after 400-h testing, (**b**) after 10,000-h testing and 1 g/m^2^ chloride (**c**) after 400-h testing, and (**d**) after 10,000-h testing at 70% relative humidity.

SEM micrographs of Figure 10 reveal corrosion bands on the specimens with chloride concentration of 0.1 g/m^2^ and 1 g/m^2^, when exposed to 70% relative humidity for 400 and 10,000 h. Figure 10a–d present the SEM micrographs of corrosion bands of Figure 2, Figure 3, Figure 6 and Figure 7 at point A, respectively. Corrosion bands are a unique corrosion feature of the specimens exposed to 70% relative humidity, not occurring with the specimens tested at 45% and 55% RH. Figure 10a,c show corrosion bands present on the 400-h tested specimens with chloride concentration of 0.1 g/m^2^ and 1 g/m^2^, respectively. For the specimens deposited with the same amounts of chloride, the number of corrosion bands on the specimen increases with increasing the test time, as illustrated by Figure 10a,b for the specimens deposited with 0.1 g/m^2^ chloride and by Figure 10c,d for those with 1 g/m^2^ chloride deposit after testing for 400 and 10,000 h, respectively. A comparison of Figure 10b,d further illustrates that the width of the corrosion band increases with increasing the chloride concentration of the specimen.

### 3.2. Analysis of EDS and EBSD

**Figure 11:**
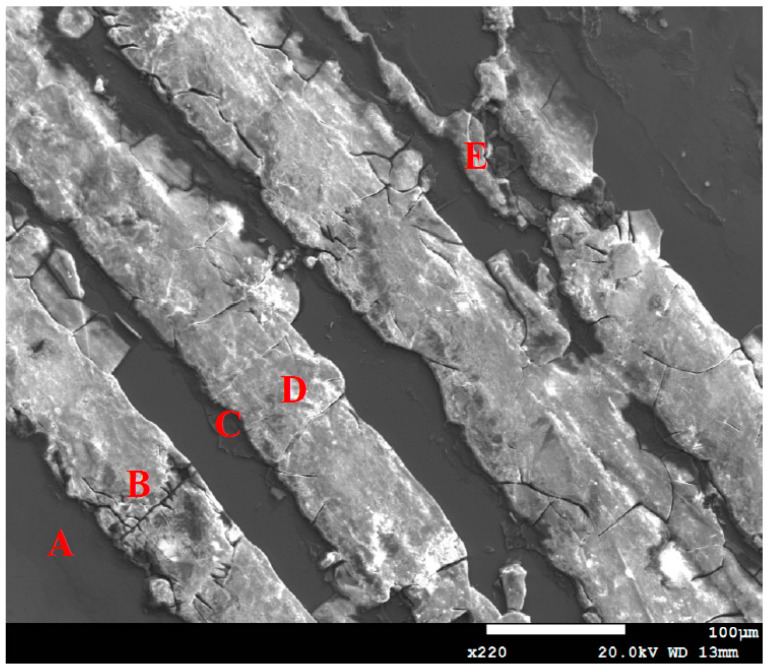
Energy-dispersive spectrometry (EDS) analysis of corrosion bands on the specimen with 1 g/m^2^ chloride deposit after testing at 70% relative humidity for 10,000 h (EDS analysis results of Points A–E are listed in Table 3).

Figure 11 depicts EDS examinations of corrosion bands on the specimen deposited with 1 g/m^2^ chloride after testing for 10,000 h tests at 70% relative humidity. The EDS analysis results of corrosion bands are listed in Table 3. The chlorine and sulfur contents of Point A, the stainless-steel matrix, are too low to be detected. Points B, D, and E, located in the corrosion bands, were analyzed to have higher chlorine contents, and the oxide at Point C to contain a trace amount of chlorine. Furthermore, Points B, C, D, and E have higher S contents, compared to Point A, which may be accounted for by sulphates contained in synthetized sea water. Moreover, the weight % of Fe and O contents at Points B and D, located in the corrosion bands, were used to identify the possible empirical formula of corrosion products. It is hypothesized that the corrosion products may be Fe(OH)_2_.

Cl^−^ ion is indispensable for the crevice corrosion initiation. The critical crevice solution mechanism ascribes the crevice corrosion initiation to the aggressive ions accumulation, particularly Cl^−^ at the crevice sites, resulting in the highly aggressive localized corrosion appearance that destroys the stainless steel passive film [27]. Points B and D have much higher Cl and O contents compared to Point C, which implies that the Cl^−^ ion may be accumulated more rapidly at those points to initiate crevice corrosion and form corrosion bands at the highest RH (RH = 70%). It is hypothesized that due to a low chloride concentration of the specimens tested, a higher relative humidity environment (RH = 70%) facilitate the transportation of chlorine (Cl) to a longer distance in straight lines to result in the linearity of the corrosion products. Therefore, corrosion bands were formed at local favorable sites and under favorable higher relative humidity environment conditions.

**Figure 12:**
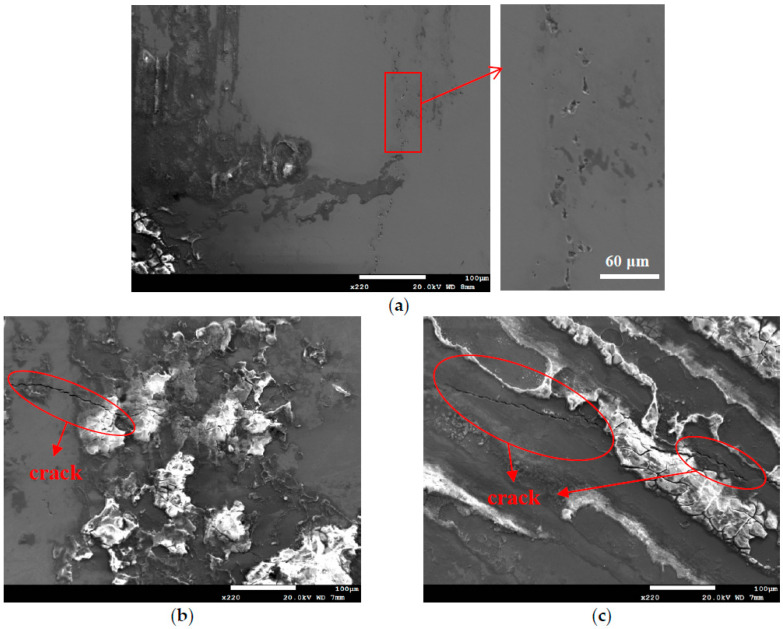
SEM morphology of stress corrosion cracks in the specimens with 1 g/m^2^ chloride deposit after 10,000-h testing at: (**a**) RH = 45%, (**b**) RH = 55%, and (**c**) RH = 70%.

**Figure 13:**
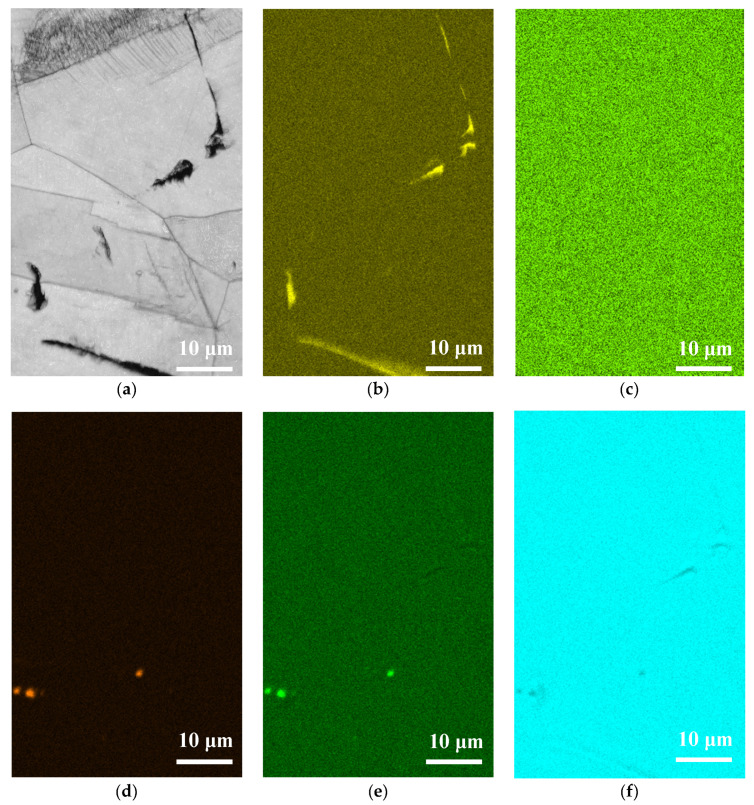

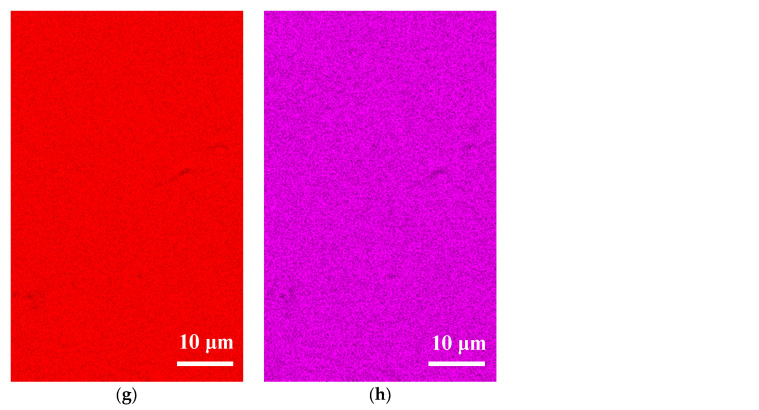
EDS mapping for the crack regions of the specimens with 1 g/m^2^ chloride concentration tested for 10,000 h at RH = 45%: (**a**) Band contrast image, (**b**) O mapping, (**c**) Cl mapping, (**d**) S mapping, (**e**) Mn mapping, (**f**) Fe mapping, (**g**) Cr mapping, and (**h**) Ni mapping.

**Figure 14:**
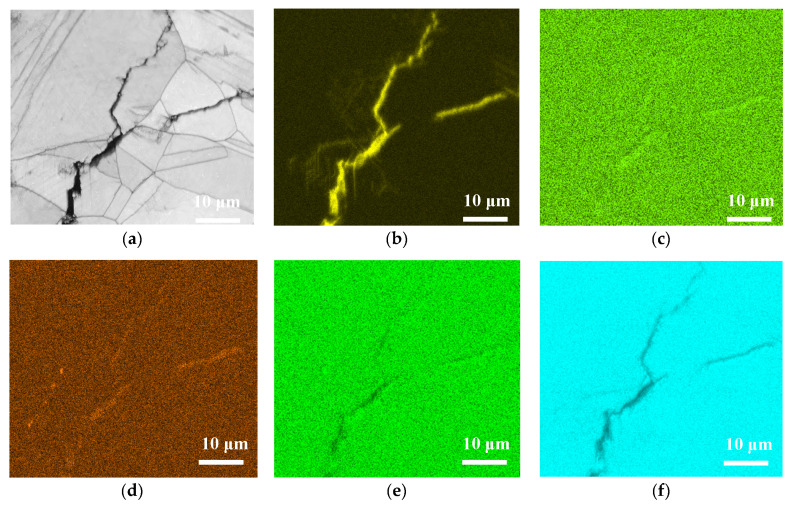

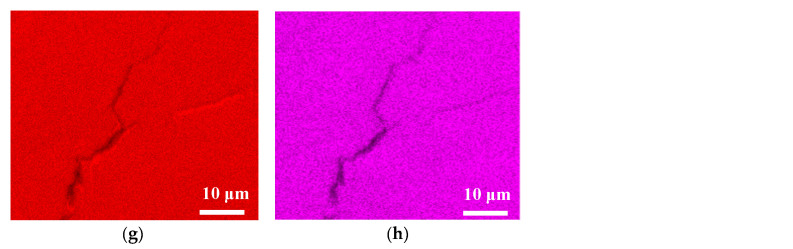
EDS mapping for the crack regions of the specimens with 1 g/m^2^ chloride concentration tested for 10,000 h at RH = 55%: (**a**) Band contrast image, (**b**) O mapping, (**c**) Cl mapping, (**d**) S mapping, (**e**) Mn mapping, (**f**) Fe mapping, (**g**) Cr mapping, and (**h**) Ni mapping.

**Figure 15:**
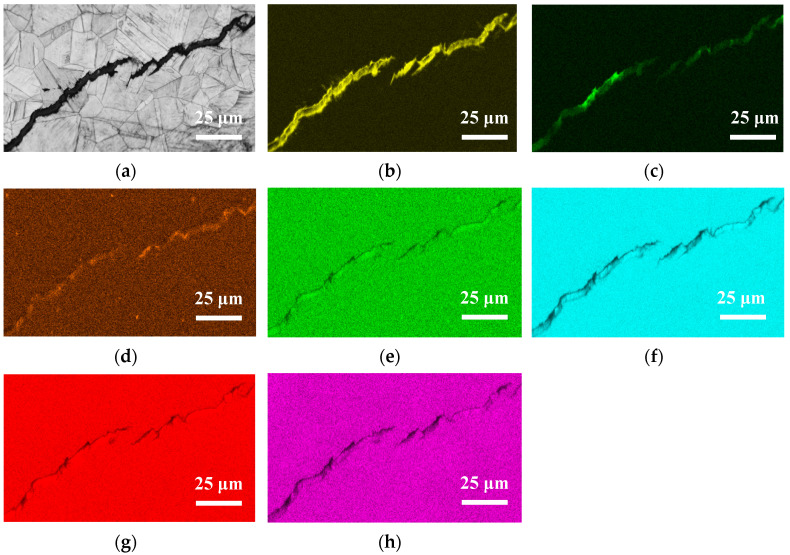
EDS mapping for the crack regions of the specimens with 1 g/m^2^ chloride concentration tested for 10,000 h at RH = 70%: (**a**) Band contrast image, (**b**) O mapping, (**c**) Cl mapping, (**d**) S mapping, (**e**) Mn mapping, (**f**) Fe mapping, (**g**) Cr mapping, and (**h**) Ni mapping.

Figure 12 is the SEM micrographs revealing the morphology of stress corrosion cracking of the specimens deposited with 1 g/m^2^ chloride after 10,000-h tests at different levels of relative humidity. Figure 12a shows discontinuous SCC cracks on the specimen exposed to 45% relative humidity, whereas Figure 12b,c demonstrate distinct SCC cracks on the specimens tested at RH = 55% and 70%. Moreover, Shoji et al. presented that MgCl_2_ is the main sea-salt constituent for the promotion of low temperature atmospherically-induced stress corrosion cracking in type 316L and 304L steels. It was investigated that the AISCC phenomenon of specimens deposited with a chloride concentration of 25 g/m^2^ by sea water [19]. Prosek et al. examined AISCC phenomenon of type 304 and 316L steel with a chloride concentration of 260 g/m^2^ obtained by MgCl_2_ droplets [20]. The chloride concentration of 1 g/m^2^ used in this work is much lower than those reported in the literature [19,20,21]. Figure 12a shows short and shallow cracks on the specimens tested at 45% RH, as opposed to Figure 12b,c, long and deep cracks are present on those exposed to 55% and 70% RH. It is hypothesized that microcracks first nucleate discretely at favorable sites and, under favorable conditions, microcracks grow and then coalesce by breaking the ligaments between them into main cracks [24,25,28,29]. Based on the hypothesis, few short and shallow cracks observed on the specimens with chloride concentration of 1 g/m^2^ at RH = 45%, the lowest RH of the three, could be accounted for by a limited amount of chloride transported to the crevice sites of the specimens. Due to a low chloride concentration of the specimens tested, it is necessary to have a higher relative humidity environment to facilitate the transportation of a sufficient amount of chlorine (Cl) to the crevice sites for crack nucleation. The chlorine (Cl) maps by EDS mapping, as shown in Figure 13c, Figure 14c and Figure 15c, are the evidence that could substantiate the aforementioned argument. It will be discussed in detail later on. However, no cracks were observed at three different relative humidity levels with the specimens deposited with chloride concentration of 0.1 g/m^2^, as illustrated in Figure 5. It could be that the 0.1 g/m^2^ chloride concentration was too low for the initiation of SCC in the specimens examined at the pre-set RH levels. From the results of this study, it is inferred that the chloride concentration threshold for the initiation of SCC for 304L steel at 45 °C is between 0.1 g/m^2^ and 1 g/m^2^. The increase of corrosion products volume could result in local stresses beneath the crevice former to prompt the happening of stress corrosion cracking. Moreover, crack length was observed to increase with the increasement of RH when comparing Figure 12b–c.

Figure 13, Figure 14 and Figure 15 respectively present the EDS mapping for the crack regions of the specimens deposited with chloride concentration of 1 g/m^2^ tested at RH = 45%, 55%, and 70% for 10,000 h. For the specimen exposed to 45% RH, the crack region is apparently enriched with oxygen, Figure 13b, but depleted with iron, Figure 13f. It may be explained that the corrosion products of iron were formed near the crack region, leading to the iron depletion at the crack region. Figure 13d,e respectively indicate enriched sulfur and manganese on the center and left bottom of the figure. The distribution of sulfur and manganese is only associated with the presence of non-metallic inclusions of MnS in the metal, and it have nothing to do with the crack. No indication of chlorine is discerned in the crack region of the specimen tested at 45% RH, as manifested in Figure 13c. For the specimens tested at 55% and 70% RH, it is found that more oxygen, chlorine and sulfur are enriched in the crack regions, as respectively demonstrated by Figure 14b–d and Figure 15b–d, but that manganese, iron, are depleted, as manifested by Figure 14e,f and Figure 15e,f. It is also found that chromium is enriched near the crack regions, as respectively demonstrated by Figure 14g and Figure 15g. Nickel is enriched near the crack regions, as manifested in Figure 15h. Moreover, a comparison of Figure 14c and Figure 15c further shows that the concentration of chlorine in the crack region significantly increases with increasing the relative humidity from 55% to 70% RH.

**Figure 16:**
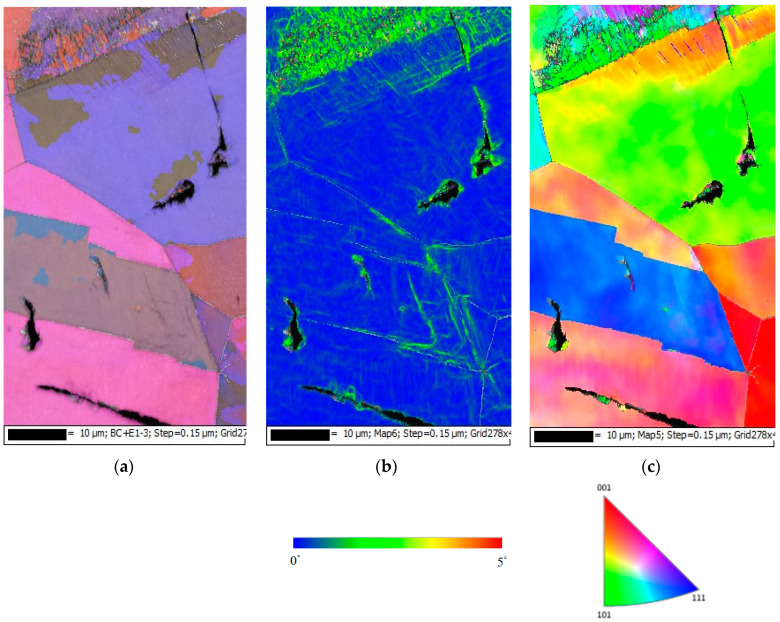
EBSD maps of the crack region of the specimen deposited with 1 g/m^2^ chloride concentration tested for 10,000 h at RH = 45%: (**a**) Euler map, (**b**) kernel average misorientation (KAM) map, and (**c**) inverse pole figure (IPF) map.

**Figure 17:**
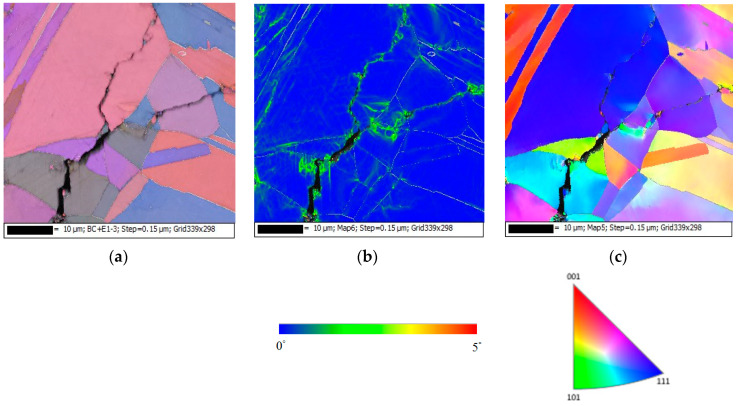
Electron backscatter diffraction (EBSD) maps of the crack region of the specimen deposited with 1 g/m^2^ chloride concentration tested for 10,000 h at RH = 55%: (**a**) Euler map, (**b**) KAM map, and (**c**) IPF map.

**Figure 18:**
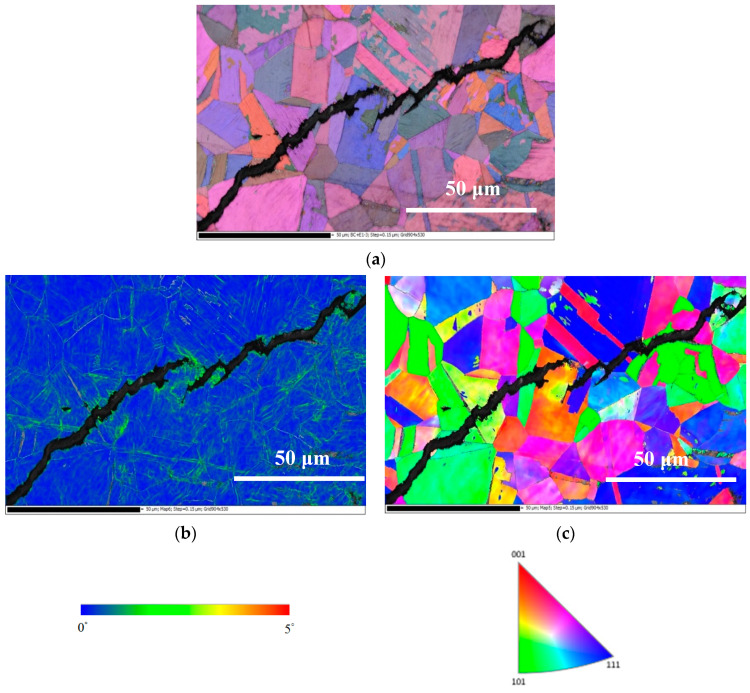
EBSD maps of the crack region of the specimen deposited with 1 g/m^2^ chloride concentration tested for 10,000 h at relative humidity of 70%: (**a**) Euler map, (**b**) KAM map, and (**c**) IPF map.

Figure 16, Figure 17 and Figure 18 present the EBSD maps of the crack region of the specimen deposited with 1 g/m^2^ chloride tested for 10,000 h at RH = 45%, 55% and 70%, respectively. Figure 16a,c, Figure 17a,c and Figure 18a,c demonstrate that the specimens were cracked by a trans-granular SCC (TGSCC) mode, which is in excellent agreement with the observations on TGSCC of stainless steel caused by chlorides at temperatures above 50 °C [11]. The kernel average misorientation (KAM) maps for specimens tested, Figure 16b, Figure 17b and Figure 18b, reveal high plastic strain around crack regions that could be related to plastic strain at the crack tip. The crack propagation was related to plastic strain at the crack tip. Although the discrete SCC cracks were only observed with specimens exposed to 45% RH, as shown in Figure 16, higher plastic strain was around crack region, and the crack mode of SCC is still TGSCC, similar to the continuous SCC cracks with specimens exposed to 55% and 70% RH, as respectively demonstrated by Figure 17 and Figure 18.

## 4. Conclusions

304L steel crevice corrosion behaviors were investigated by testing the specimens at 45 °C under a combination of the chloride concentration of 0.1 and 1 g/m^2^, the relative humidity of 45%, 55% and 70% and the test duration of 400 h and 10,000 h in this study. The effects of the chloride concentration and relative humidity on the corroded regions were evaluated and a chloride concentration threshold for the SCC initiation according to the test results was proposed. The chloride concentration of 0.1 and 1 g/m^2^ used in this study differs from the concentration of salts in seawater. The conclusions of this study are given as below:

The specimens with chloride concentration of 0.1 g/m^2^ had minimum corrosion areas at relative humidity of 55%, and there were no cracks at three different relative humidity levels. It may be that the concentration of 0.1 g/m^2^ deposit chloride was too low for the initiation of SCC.

(1) The specimens with chloride concentration 1 g/m^2^ tested for 400 h at 55% RH had minimum rusted areas. Nevertheless, there was little difference in the rusted areas of the specimens tested at 45% and 55% RH for 10,000 h, whereas the area of rust significantly increased when the RH increased to 70%. Some shallow corrosion beneath rust spots were found, but no cracks were observed in the corrosion region after 400-h testing, whereas SCC cracks were observed with the specimens tested for 10,000 h at all relative humidity levels. The discrete SCC cracks were observed with specimens exposed to 45% RH, but, on the other hand, distinct stress corrosion cracking cracks were found with those tested at 55% and 70% RH. This can be accounted for by a limited amount of chloride transported to the sites of crevice of the specimens with chloride concentration of 1 g/m^2^ exposed to 45% RH, the lowest RH. It may be necessary to have a higher RH environment to facilitate transportation of chloride to the crevice sites, because of the low chloride concentration of the specimens. The observations that the chlorine concentration at the crack regions increases with increasing the relative humidity give evidence to substantiate the aforementioned argument.
(2) The results of the 10,000-h tests of this research suggest that the chloride concentration threshold for the initiation of SCC in 304L stainless steel at 45 °C is between 0.1 g/m^2^ and 1 g/m^2^.
(3) Corrosion bands were exclusively observed on the specimens with chloride concentration of 0.1 g/m^2^ and 1 g/m^2^ tested at 70% RH. The width of corrosion band increased with increasing chloride concentration. Moreover, corrosion bands were analyzed to have higher chlorine contents.
(4) The 304L austenitic stainless steel specimens with chloride concentration of 1 g/m^2^ tested at 45 °C were cracked with a trans-granular SCC mode, which was proved by the results of EBSD.

## Figures and Tables

**Table 1:** Chemical compositions of 304L stainless steel used in this work.

Element	C	S	Si	Ni	Cr	Mn	Fe
wt.%	0.017	0.0290	0.450	9.000	18.000	1.540	Bal.

**Table 2:** Chemical compositions of sea salt used in this work.

Composition	NaCl	MgCl_2_	Na_2_SO_4_	CaCl_2_	KCl	NaHCO_3_	KBr	SrCl_2_	H_3_BO_3_	NaF
wt%	58.490	26.460	9.750	2.765	1.645	0.477	0.238	0.095	0.071	0.007

**Table 3:** EDS analysis results of corrosion bands (wt.%).

Location	O	Na	Mg	S	Cl	Ca	Cr	Mn	Fe	Ni
A	0.73	0.11	0.04	0.00	0.00	0.04	18.78	1.74	70.46	8.10
B	33.2	0.98	0.30	0.24	3.11	0.06	8.67	0.38	51.77	1.28
C	16.59	1.64	0.60	0.06	0.31	0.08	16.83	1.69	56.05	6.14
D	30.31	1.26	0.62	0.18	2.98	0.17	13.48	0.87	48.51	1.63
E	27.74	0.36	0.45	0.16	2.40	0.09	6.47	0.96	57.95	3.42
